# A Structural View of Influenza Virus Ribonucleoprotein Complex and Its Functions

**DOI:** 10.3390/microorganisms14071486

**Published:** 2026-07-07

**Authors:** Yixiao Liu, Lejin Zhang, Yuqi Lin, Zhiyong Lou

**Affiliations:** 1 School of Basic Medical Sciences, Tsinghua University, Beijing 100084, China; 2 Division of Life Sciences and Medicine, University of Science and Technology of China, Hefei 230026, China

**Keywords:** influenza virus, viral ribonucleoprotein complex, RNA polymerase, nucleoprotein, cryo-electron microscopy, antiviral drug development

## Abstract

Influenza viruses are a major global health threat because of their recurring seasonal burden and continuing pandemic potential. Central to the viral life cycle is the viral ribonucleoprotein complex (vRNP), the functional unit of the segmented genome, in which each negative-sense RNA segment is encapsidated by oligomeric nucleoprotein (NP) and bound at its termini by the polymerase complex (FluPol). Recent advances in structural biology have revealed high-resolution structures of FluPol in distinct conformations, NP-RNA helical assemblies, and intact vRNP architectures, providing a structural framework for understanding vRNP assembly, polymerase conformational switching, and RNA synthesis in the RNP context. By contrast, current models for vRNP trafficking and selective genome packaging still rely largely on virological, biochemical, and cell biological evidence, with only limited structural resolution. In this review, we synthesize current knowledge of vRNP assembly, transcription, replication, intracellular trafficking, and selective genome packaging, and discuss the major unresolved questions in each area as well as their implications for antiviral development.

## 1. Introduction

Influenza viruses are major human pathogens responsible for seasonal epidemics that cause substantial morbidity, mortality, and economic burden worldwide each year [1,2]. Beyond this recurring burden, pandemic influenza poses a less predictable threat, arising when antigenically novel viruses are transmitted to humans from animal reservoirs, as occurred in 1918, 1957, 1968, and 2009. The continued spread of highly pathogenic H5N1 avian influenza among wild birds, poultry, and mammals has further heightened concern about future emergence. Vaccines and antiviral drugs are essential but imperfect countermeasures against influenza [3]. Antigenic drift in hemagglutinin necessitates frequent vaccine reformulation [4,5], while resistance to antivirals, from adamantanes to the polymerase inhibitor baloxavir marboxil [6], remains a persistent clinical concern. These limitations underscore the need for a deeper understanding of how influenza viruses replicate.

Influenza A and B viruses possess eight segments of negative-sense viral RNA, whereas influenza C and D viruses contain seven [7,8]. These genomic RNAs do not exist as free molecules within virions or infected cells but are assembled into viral ribonucleoprotein (vRNP) complexes [9,10], each comprising a single vRNA segment encapsidated by multiple copies of nucleoprotein (NP) and bound at its 5′ and 3′ termini by a single copy of the heterotrimeric viral polymerase (FluPol), comprising subunits PB1, PB2, and PA [11,12,13]. The vRNP therefore represents the functional form of the viral genome. Following virus entry, vRNPs are imported into the nucleus to serve as templates for mRNA transcription and genome replication, and newly assembled vRNPs are subsequently exported and trafficked to the plasma membrane for selective incorporation into budding virions [11,14,15].

Structural studies of isolated FluPol and NP have provided essential foundations for understanding vRNP function. Atomic-resolution structures of FluPol revealed the conformational states underlying cap-snatching, transcription, and the transition to replication [16,17,18,19,20,21]. Crystal structures of NP oligomers established the tail-loop contacts mediating NP oligomerization [22], while structures of reconstituted NP-RNA filaments revealed the molecular basis of sequence-nonspecific RNA binding [23,24]. Despite these advances, the structural organization of the intact vRNP has remained considerably more difficult to characterize, owing to the inherent flexibility and heterogeneity of the complex. More recently, cryo-EM structures of reconstituted mini-vRNP systems captured the atomic-resolution interface between FluPol and the NP-RNA scaffold [25], whereas combined cryo-EM and cryo-ET analyses of full-length reconstituted vRNPs established the right-handed double-helical organization of the complex and provided the first structural views of polymerase engagement with the helical template during RNA synthesis [26].

These structural foundations have informed mechanistic studies of vRNP function throughout the viral life cycle, yet structural characterization of the intact vRNP in its various functional states is still an ongoing challenge. The molecular basis of vRNP nuclear import, export, and cytoplasmic transport has been substantially clarified through identification of the host factors and post-translational modifications that regulate each step, while structural information for vRNP-host factor complexes at key trafficking checkpoints is limited [11,15]. Similarly, the problem of selective genome packaging has advanced through identification of segment-specific packaging signals and mapping of inter-segment RNA-RNA contacts [27]. Although the overall arrangement of the eight vRNP segments within the virion has been established by cryo-ET [28], the molecular basis of the inter-segment contacts that mediate selective packaging has not been elucidated. On the antiviral side, the clinical approval of baloxavir marboxil has validated FluPol as a druggable target, but the rapid selection of resistance variants underscores the need to identify and structurally characterize additional vulnerable sites within the vRNP machinery [29,30].

**Figure 1:**
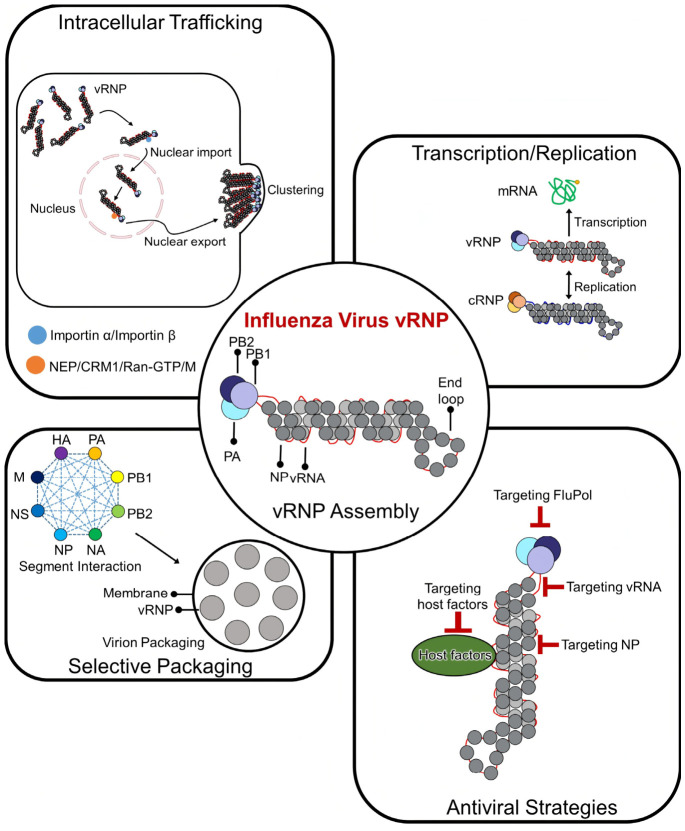
Integrated schematic of influenza vRNP organization, function, and antiviral targeting. This diagram summarizes vRNP architecture and functional mechanisms together with major antiviral targeting strategies. Left panels depict intracellular trafficking of vRNPs, including nuclear import, export, and cytoplasmic clustering. Upper right illustrates transcription and replication of vRNPs. Center shows the structural organization of the vRNP composed of PB2, PB1, PA, NP, and vRNA. Lower left summarizes selective packaging of the eight genomic segments via inter-segment interactions. The blue dotted line represents possible interactions between segments. Lower right highlights major antiviral strategies targeting FluPol, NP, vRNA elements, and host factors involved in vRNP regulation. The red T-shape represents the inhibitory effect of the inhibitor.

This review focuses on how structural organization shapes vRNP function across the influenza virus life cycle. We examine current understanding of vRNP architecture and assembly, the basis of transcription and replication in the RNP context, and the emerging mechanistic frameworks for intracellular trafficking, selective genome packaging, and antiviral targeting (Figure 1).

## 2. Overall RNP Assembly Architecture

**Figure 2:**
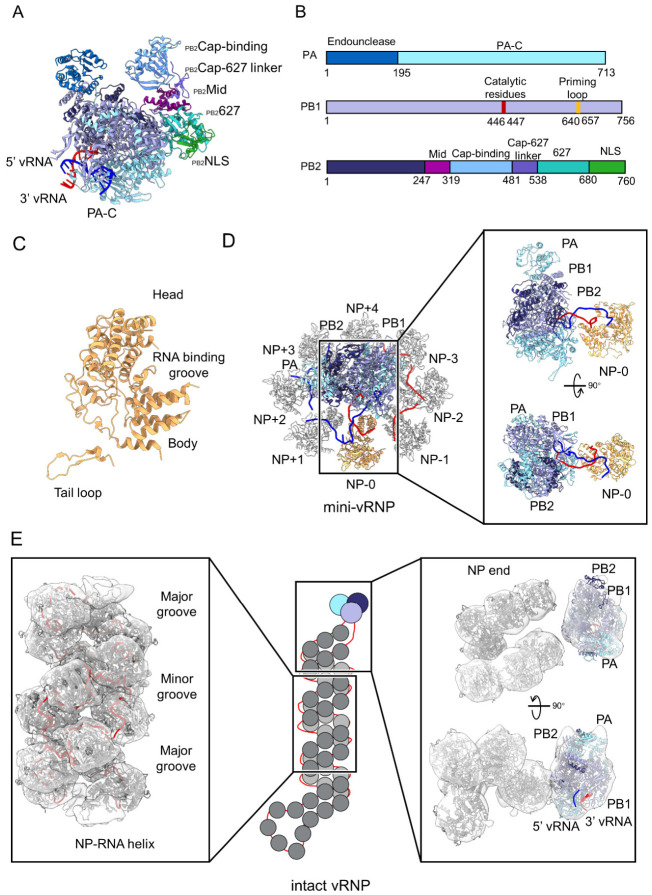
Structures of influenza virus RNP. (**A**) Crystal structure of the bat influenza A virus FluPol (PDB:4WSB [16]), displayed in cartoon style with components and domains labeled in different colors. The N-terminal exonuclease domain of PA is shown in dark blue, PA-C in cyan, PB1 in violet, PB2-N in navy, _PB2_Mid in purple, _PB2_Cap-binding in light blue, _PB2_Cap-627 linker in blue-purple, _PB2_627 in teal, _PB2_NLS is green, the 5′ vRNA promoter in blue, and the 3′ vRNA promoter in red. (**B**) Schematic diagram showing the distribution of domains within the three subunits (PA, PB1, PB2) of FluPol, corresponding to (**A**). The start and end residues of each domain within the subunits are labeled below the diagram. The color scheme for each domain is consistent with that in (**A**). The catalytic residues and priming loop in PB1 are labeled in red and yellow, respectively. (**C**) The crystal structure of the NP protein of the influenza A virus is displayed in cartoon style (PDB:2IQH [22]), with the names of each domain labeled at their corresponding positions in the structure. (**D**) The overall structure of the mini-vRNP is shown in cartoon style [25]. Within the FluPol complex, the PA, PB1, and PB2 subunits are colored in light blue, violet, and navy, respectively. NP-0 is shown in gold, while NPs at regular positions are colored in white. The 5′ vRNA is colored in blue, and the 3′ vRNA in red. The right panels display magnified views of the FluPol:RNA:NP-0 structural unit from two orthogonal perspectives, illustrating how NP-0 binds the RNA duplex and facilitates strand separation. (**E**) Schematic representation of the overall architecture of the full-length NS segment vRNP derived from influenza D virus. The central panel presents a schematic diagram of the complete structure. The left panel highlights the double-helical organization of the NP-RNA assembly within the intact RNP (PDB:9C4H [26]), displayed in cartoon representation, with the two NP filaments colored in gray and white, respectively, and the associated RNA strand in red. The corresponding cryo-EM density is shown as a semi-transparent surface. The right panel depicts the terminal region of the complete RNP containing the FluPol complex, with the cryo-EM density shown as a semi-transparent surface. The atomic models of FluPol and NP are docked into their respective densities and displayed in cartoon style. NP is colored in white, and the color scheme for FluPol is consistent with that in (**C**).

At the heart of each vRNP is FluPol, a ~250 kDa complex of subunits PB1, PB2, and PA, each contributing distinct functional domains [12,13]. PB1 contains the RNA-dependent RNA polymerase active site with the catalytic SDD motif responsible for phosphodiester bond formation [16,31]. PA comprises an N-terminal endonuclease domain that cleaves capped host pre-mRNAs to generate transcription primers [32,33,34], while PB2 contains a cap-binding domain that recognizes 5′ cap structures of host mRNAs, as well as the 627 and NLS domains that contribute to host adaptation and nuclear import, respectively. The three subunits assemble through conserved interfaces in which the PB1 N-terminus inserts into a hydrophobic pocket in the PA C-terminal domain and the PB1 C-terminus contacts the PB2 N-terminal region [35,36,37] (Figure 2A,B). The first atomic-resolution structure of FluPol bound to the vRNA promoter revealed that the 5′ and 3′ termini form a partially complementary panhandle, with the 5′ end folding into a compact hook lodged at the PA-PB1 interface and the 3′ end bound at a composite site formed by residues from PB1, PB2, and PA [16] (Figure 2A). Between the transcriptase and replicase conformations, the PA endonuclease domain, PB2 cap-binding domain, and 627 domain undergo large-scale repositioning, allowing a single polymerase to execute two mechanistically distinct modes of RNA synthesis [17,18,19,20,21,38,39].

NP is the most abundant protein component of the vRNP, present at approximately one copy per 24 nucleotides of vRNA [11,40]. Crystal structures from multiple influenza subtypes reveal a crescent-shaped monomer with head and body domains flanking a positively charged groove that constitutes the RNA-binding surface [22,41,42,43] (Figure 2C). This groove enables sequence-nonspecific recognition of single-stranded RNA, consistent with the requirement for NP to encapsidate all eight genomic segments regardless of sequence [22,43,44]. NP oligomerises through a flexible C-terminal tail loop that inserts into a hydrophobic pocket on an adjacent monomer; mutations disrupting this interface abolish both NP oligomerization and RNA synthesis activity, confirming that tail-loop mediated NP-NP interaction is essential for vRNP assembly [22,45,46,47]. Near-atomic resolution cryo-EM structures of reconstituted NP-RNA filaments have revealed the path of RNA along the positively charged groove, with NP monomers linked head-to-tail through tail-loop contacts [23,24].

Determining the structure of intact vRNPs has proven considerably more challenging. Two general strategies have been employed. Native vRNPs can be extracted from virions, preserving authentic composition but suffering from inherent flexibility and length heterogeneity across the eight genomic segments; these have limited cryo-EM reconstructions to nanometer resolution and precluded detailed analysis of the FluPol-NP interface [48,49]. Reconstitution from recombinant components has partially overcome these obstacles.

Mini-vRNP systems employ co-expression of FluPol, NP, and a truncated vRNA in mammalian cells, yielding closed circular complexes that retain transcription and replication activity [40,50,51,52]. Recent cryo-EM analysis of such complexes achieved near-atomic resolution, revealing the molecular coupling between FluPol, NP, and RNA within an assembled mini-vRNP [25]. This work revealed that a single NP monomer, designated NP-0, makes the primary contact with FluPol through interactions between its head domain and the PA and PB1 subunits (Figure 2D). The structure further showed how the distal vRNA promoter duplex is separated at the FluPol-NP junction, establishing the structural basis for polymerase anchoring and RNA organization within the complex. Mini-vRNPs, however, form circular structures and lack full-length viral RNA, leaving questions concerning the overall architecture of native rod-shaped vRNPs unresolved.

Full-length vRNP reconstitution has yielded complementary insights. Using the influenza D virus NS segment, the shortest genomic RNA and thus the one yielding the most conformationally homogeneous vRNP particles, a recent study combined cryo-EM with cryo-ET to resolve the double-helical structure and visualize the polymerase within the intact complex [26] (Figure 2E). The resulting reconstruction confirmed a right-handed anti-parallel helical architecture, with vRNA encapsidated in the minor groove and adjacent NP subunits connected through the conserved tail loop. Cryo-ET analysis further captured the polymerase at multiple positions along the helix, suggesting that FluPol can translocate relative to the NP-RNA scaffold during RNA synthesis while maintaining the overall double-helical organization. The polymerase region, however, was resolved only to 20–30 Å, insufficient for detailed characterization of FluPol-NP contacts in the full-length context.

These two approaches provide complementary perspectives, with mini-vRNPs offering high-resolution detail of the FluPol-NP-RNA interface and full-length vRNPs revealing overall helical organization and polymerase dynamics along the native template. Key questions remain, most notably the detailed molecular interactions between FluPol and NP within the context of full-length vRNPs and whether conclusions drawn from the shortest segment can be generalized to longer, more flexible vRNPs. These structures capture assembled end-states rather than the process by which progeny vRNPs form. Maintaining a proper balance between monomeric and oligomeric NP, together with subsequent RNA binding, is critical for correct vRNP assembly. Phosphorylation-dependent control of the NP-NP interaction has been proposed to maintain a pool of monomeric, RNA-competent NP [53], whereas host factors such as UAP56 may further regulate NP assembly states by coordinating NP oligomerization and RNA binding [54,55]. During replication, ANP32 further contribute to vRNP assembly by bridging FluPol and promoting NP recruitment to nascent RNA [39,56]. However, how these sequential events are coordinated in infected cells remains poorly understood. These observations suggest that progeny vRNP assembly is not a simple spontaneous process but rather a regulated pathway involving multiple transient intermediate states that remain structurally unresolved.

## 3. Transcription and Replication Mechanisms of the RNP

The vRNP is the direct functional unit for influenza virus genome transcription and replication. Throughout the viral life cycle, the polymerase remains stably associated with both termini of the vRNA, and it is within this nucleoprotein-coated context that transcription and replication occur. A central question is therefore how the polymerase accesses the entire length of the template without disrupting the double-helical architecture. Equally important is how the vRNP coordinates the transition between transcription and replication as infection proceeds.

During transcription, the vRNP-associated polymerase acquires capped primers from nascent host pre-mRNAs via cap-snatching [17,18,32], then synthesises viral mRNA processively along the encapsidated vRNA template. Progression through the transcription cycle is accompanied by a series of conformational changes within the polymerase. The PB2 cap-binding domain recognizes host mRNA caps and undergoes repositioning to deliver the substrate to the PA endonuclease active site, which cleaves the mRNA 10–13 nucleotides downstream to generate a capped primer for viral RNA synthesis [17,33]. The PB1 priming loop is displaced during elongation to accommodate the growing product-template duplex [19,38]. Throughout elongation, the template 3′ end is held at a secondary binding site between PA-C and the PB1 palm domain, while the 5′ hook remains anchored within the polymerase. Elongation terminates by reiterative stuttering at the 5′-proximal oligo(U) signal, generating the poly(A) tail [19]. Product release is followed by a conformational reset toward the pre-initiation state, allowing successive rounds of synthesis [19]. Because FluPol associates with both vRNA termini throughout RNA synthesis, template translocation cannot occur through simple threading of RNA through a stationary polymerase. Instead, movement of the template relative to the polymerase requires coordinated conformational rearrangement of the entire vRNP [19]. Negative-stain EM of vRNPs under transcription-activating conditions revealed FluPol at internal positions along the NP-RNA helix rather than exclusively at the terminus, indicating displacement of the polymerase relative to the helical filament [49]. In the same study, nucleozin-mediated crosslinking of adjacent NP subunits, which locks helix flexibility, substantially inhibited transcription, providing functional evidence that inter-strand sliding is required for processive synthesis [49]. These observations gave rise to a processive helical track model, in which the two anti-parallel NP-RNA strands slide past one another, allowing the polymerase to move along the genome while remaining tethered to both termini and without disrupting helical architecture.

HS-AFM and cryo-EM analysis of virion-derived vRNPs during in vitro RNA synthesis provided direct evidence that helical scaffold integrity is a prerequisite for processive RNA synthesis [57]. vRNPs that maintained the intact helical rod-shaped architecture were associated with folded single-stranded nascent RNA, consistent with productive synthesis via the helical track mechanism. In contrast, structurally deformed vRNPs exhibiting loss of helical grooves and reduced mechanical stability were associated with looped double-stranded RNA that had partially dissociated from NPs, indicative of abortive synthesis. single-molecule HS-AFM imaging of reconstituted mini-vRNPs captured the conformational cycle in real time, demonstrating that individual complexes can complete consecutive rounds of synthesis without disassembly [58].

**Figure 3:**
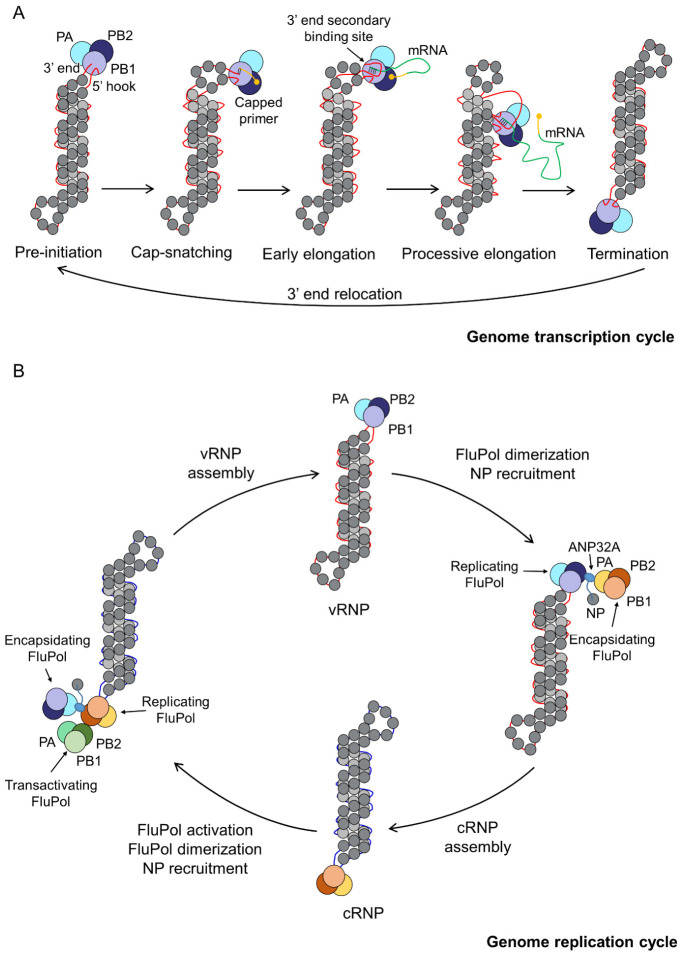
Transcription-replication mechanisms in the context of RNP (**A**) Schematic illustration of the “strand-sliding” model during vRNP transcription, depicting the structural configurations of the RNP at four sequential stages: transcription initiation, early elongation, processive synthesis, and termination. Throughout the transcription cycle, both the 3′ and 5′ termini of the vRNA template remain tethered to the polymerase complex, thereby maintaining the overall double-helical architecture of the RNP. (**B**) Schematic of the vRNP replication cycle, illustrating the coordinated interplay among the replicating FluPol, the encapsidating FluPol, and the transactivating FluPol during both the vRNP-to-cRNP and the cRNP-to-vRNP replication processes.

Complementing these 2D observations, cryo-ET subtomogram averaging of reconstituted influenza D vRNPs captured the polymerase at distinct positions along the NP-RNA filament, with rearrangements of the surrounding NP subunits, supporting a strand sliding model in which processive RNA synthesis proceeds without disrupting the overall double-helical architecture [26] (Figure 3A). These observations suggest that processive RNA synthesis requires coordinated movements of FluPol and the NP-RNA scaffold, although the molecular details of how polymerase rearrangements are coupled to helix dynamics remain unresolved.

Replication differs fundamentally from transcription in that it initiates de novo without a capped primer and proceeds through two sequential steps: parental vRNA is first copied into positive-sense cRNA, which then serves as template to produce progeny vRNA [20,39,59,60]. Replication of the influenza genome requires the cooperation of multiple FluPol molecules assembled into distinct complexes at each replication step. In both replication directions, ANP32 bridges the replicating FluPol with an encapsidating FluPol to form an asymmetric dimer, and the LCAR of ANP32 recruits free NP to the replication complex, enabling progressive NP encapsidation of the nascent RNA strand during daughter RNP assembly [39,56,59,61,62]. For vRNA to cRNA synthesis, this ANP32-bridged replicase-encapsidase dimer is sufficient to carry out cRNA synthesis and co-replicational cRNP assembly [39,63]. For cRNA to vRNA synthesis, a third FluPol molecule is additionally required to activate the replicating polymerase in trans through direct FluPol-FluPol contacts, initiating vRNA synthesis from the cRNA [20,59,62] (Figure 3B). Unencapsidated replication intermediates are rapidly degraded, ensuring that only properly assembled daughter RNPs accumulate [64].

Compared with transcription, the structural basis of replication within the intact vRNP context is less well defined. Negative-stain EM of reconstituted vRNPs has identified branched structures interpreted as nascent daughter RNPs emerging from parental templates [65]. More recently, cryo-ET analysis detected vRNP complexes containing two FluPol molecules, one at the helical terminus and another bridging two vRNPs at an internal position, proposed to represent replication initiation and elongation intermediates, respectively [26]. How the replicase-encapsidase-ANP32 machinery is spatially organized relative to the parental RNP helix, and how nascent RNA is transferred from replicase to encapsidase, are questions that await high-resolution structural characterization.

The transition from transcription to replication during infection is associated with large-scale FluPol conformational rearrangements together with changes in its interactions with viral and host factors [59]. In the transcriptase conformation, the PB2 cap-binding and 627 domains adopt positions that support cap-snatching and primer-dependent RNA synthesis, whereas in the replicase conformation these domains are repositioned to support de novo initiation [13]. A transcription-replication intermediate has been described in which the PA endonuclease and PB2 cap-binding domains become partially unresolved and the polymerase core adopts a more open conformation, potentially representing a transition state between transcriptional and replicative modes [21]. The viral protein NS2/NEP, expressed predominantly at later stages of infection, binds FluPol and occludes its binding site for host RNA polymerase II, thereby preventing cap-snatching and redirecting polymerase activity toward replication [66]. The progressive accumulation of NS2/NEP as infection proceeds thus provides a mechanism for the transition from early transcription-dominated to later replication-dominated phases of the viral life cycle [67].

Collectively, these studies show that the vRNP can resolve the topological constraints of processive elongation within a double-helical scaffold and sustain multiple rounds of RNA synthesis without disassembly. The precise molecular mechanism of template recycling, how the complete replication cycle proceeds within the structural context of the intact vRNP, and how the transcription-to-replication switch is coordinated at the molecular level are important questions for future work.

## 4. Intracellular Trafficking and Host Interactions of the RNP

Unlike most RNA viruses, which replicate in the cytoplasm, influenza viruses carry out transcription and replication exclusively in the nucleus. Productive infection therefore depends on tightly coordinated vRNP trafficking; incoming vRNPs are imported into the nucleus following viral entry, and progeny vRNPs are exported to the cytoplasm for incorporation into budding virions [11,15].

**Figure 4:**
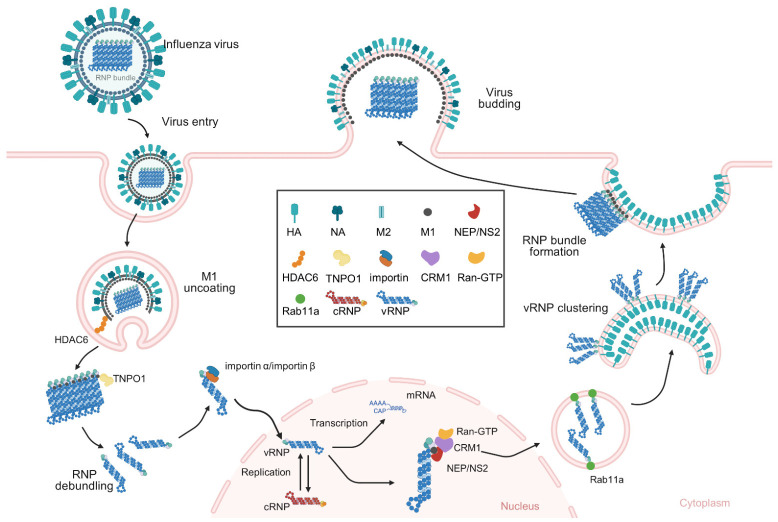
Intracellular transport pathways of RNPs This figure illustrates the intracellular trafficking pathway of RNPs. Upon entry into the host cell, vRNPs first undergo dissociation from M1 and debundling facilitated by TNPO1, followed by nuclear import via the importin α/importin β-mediated pathway. Within the nucleus, vRNPs serve as templates for both transcription and genome replication. Newly replicated vRNPs are assembled into nuclear export complexes comprising M1, NEP, CRM1, and RanGTP, which mediate their translocation through the nuclear pore complex into the cytoplasm. Upon nuclear export, vRNPs associate with Rab11a-positive recycling endosomes and are transported along microtubules toward the plasma membrane. In a Rab11a-dependent manner, vRNPs accumulate at high density on the cytoplasmic face of HA-membranes, forming localized vRNP clusters. Finally, M1 interacts with vRNPs to coordinate the final genome packaging and budding of progeny virions from the cell surface.

After viral entry and endosomal acidification, the M1 matrix layer disassembles and incoming vRNPs are released into the cytoplasm as bundles. Two host factors act sequentially to prepare these bundles for nuclear import [68]. The cytoplasmic deacetylase HDAC6 recognizes ubiquitin chains on the incoming viral particle and routes it through an aggresome-like processing pathway that facilitates uncoating. The transportin-1 (TNPO1) then binds the PY-NLS in the M1 N-terminal domain, promotes M1 removal, and induces vRNP bundle disassembly; only after debundling do individual vRNPs engage importin-α/β for nuclear import (Figure 4) [68,69,70].

Nuclear export of newly synthesized vRNPs is mediated by a daisy-chain mechanism [71]. M1 binds directly to vRNPs and recruits NEP through its NLS domain; NEP in turn recruits CRM1 via its NES motifs to form the export complex with RanGTP, and CRM1 drives translocation through the nuclear pore [71]. This pathway is subject to multi-layered post-translational regulation. Phosphorylation of a conserved S-S-S motif in the NEP N-terminus is required for CRM1 recruitment and export activity in both influenza A and B viruses [72]. Activation of the host Raf/MEK/ERK/RSK1 signaling cascade, triggered by PKCα upon HA accumulation at the plasma membrane, leads to RSK1-mediated phosphorylation of NP, creating a docking site for M1 at chromatin and initiating export complex assembly (Figure 4) [73]. Unidirectionality is enforced by an intramolecular autoinhibition mechanism within NEP [74]. The N-terminal NES domain masks the C-terminal M1-binding domain, preventing premature NEP-M1 association in the cytoplasm. CRM1 binding to the NES in the nucleus relieves this autoinhibition, restricting export complex assembly to the nuclear compartment.

Upon cytoplasmic entry, vRNPs are transported toward the plasma membrane via Rab11a-positive recycling endosomes [75]. The PB2 C-terminal domain interacts directly with the switch I region of Rab11a, the same surface used by Rab11 family-interacting proteins, providing the molecular link between vRNPs and the endosomal transport machinery [76]. This interaction is conserved across influenza A subtypes and is essential for genome delivery to assembly sites [76]. During this trafficking phase, ATG9A promotes dissociation of recycling endosomes from microtubules, a step required for the vRNP-laden endosomes to coalesce into cytosolic viral inclusions [77]. These inclusions are not classical membrane-bound organelles but membraneless biomolecular condensates that form by liquid–liquid phase separation (LLPS) [78]. Their assembly is driven by multivalent interactions among vRNPs, each vRNP is tethered through its PB2 subunit to Rab11a on the recycling-endosomal surface, and these vRNP-Rab11a units engage one another through weak, transient contacts that concentrate vRNPs into liquid droplets. Their formation is further tuned by the host factor ATG9A and by contacts with the endoplasmic reticulum, which together dictate where and when the inclusions condense and how readily they fuse and divide. Far from being static storage depots, they therefore behave as dynamic, liquid-like compartments whose properties can be modulated by the cell [77]. In situ cryo-ET has visualized the clustering step, revealing that HA-containing endomembranes serve as Rab11a-dependent platforms for vRNP clustering (Figure 4); when HA is absent, vRNPs cluster equivalently on NA-containing membranes, demonstrating that this step is membrane-assisted but glycoprotein-independent [14]. Concurrently, M1 is transported as multilayered helical cylinders of antiparallel dimers that partially dissociate in the cytoplasm, supplying subunits for subsequent matrix assembly [14,79,80]. Upon reaching the plasma membrane, interactions with M2 and negatively charged phospholipids drive the transition to a single-layer helical matrix, and M1 layer closure precedes budding neck formation [14]. Notably, the vRNP bundle forms exclusively within fully assembled virions rather than on endomembranes or at the plasma membrane, indicating that final genome packaging is imposed by the M1 helical scaffold at the budding site itself (Figure 4) [14]. Current evidence has outlined the overall route of intracellular vRNP trafficking, but the high-resolution architecture of the host–virus complexes that mediate these steps is still largely unknown, leaving the precise molecular basis of each step to be established.

Beyond its trafficking machinery, the RNP is also the principal interface at which the virus both triggers and evades innate immune surveillance. Viral RNA synthesis is a major source of the immunostimulatory ligands that the host detects. During replication the polymerase also generates aberrant, internally deleted RNAs, principally the short mini viral RNAs (mvRNAs) and the longer defective interfering RNAs (DI RNAs). Both RNAs are recognized by RIG-I through their 5-triphosphate ends [81]. The mvRNAs in particular are regarded as a physiological inducer of type I and III interferon (IFN) during influenza infection, and their abundance correlates with the cytokine dysregulation observed in highly pathogenic and pandemic strains [81].

To counter this detection, influenza virus has evolved multiple immune-evasion strategies. The best-characterised viral factor is NS1, which sequesters viral double-stranded RNA from cytosolic sensors and inhibits RIG-I activation [82,83]. Beyond this classical pathway, individual protein components of the RNP also antagonise host innate immunity through activities distinct from their roles in the polymerase complex. PB1 has been reported to promote autophagic degradation of the mitochondrial adaptor MAVS, limiting IFN production [84] whereas PB2 can target JAK1 for ubiquitin-dependent degradation to impair IFN receptor signalling and interferon-stimulated gene expression [85]. Whether these activities are exerted by free subunits or within the assembled complex remains to be established. Together with the immunostimulatory RNAs generated during replication, these findings indicate that the proteins and RNAs of the RNP are closely intertwined with the regulation of host innate immunity.

## 5. Selective Packaging of vRNP Segments

**Figure 5:**
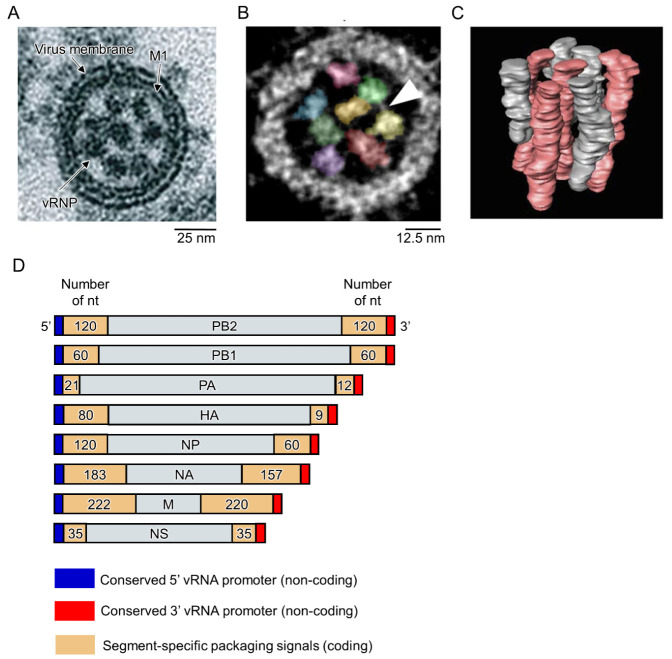
Selective packaging of the eight-segment influenza A virus genome. (**A**) The characteristic ‘7 + 1’ arrangement of vRNPs within the virion interior [86], with the viral membrane, the M1 layer, and individual vRNPs indicated by arrows, respectively. (**B**) Inter-segment interactions among the eight vRNPs within a single virion [28]. The eight RNPs colored in different colors. String-like bridging densities interconnecting adjacent vRNPs are indicated by arrowheads. (**C**) Three-dimensional reconstruction of vRNP bundle in virion [28]. The four longer vRNP segments are shown in red, and the four shorter segments in grey. (**D**) Schematic representation of the packaging signals in each of the eight influenza A virus vRNP segments. The 5′ and 3′ promoter regions of the vRNA are shown in blue and red, respectively. The segment-specific packaging signals are shown in orange, with the lengths of the 5′ and 3′ terminal coding regions required for efficient incorporation of each segment annotated in nucleotides (nt).

Selective packaging of the influenza genome is among the most precise sorting processes known in RNA virology [27]. For productive infection, progeny virions must acquire a complete set of distinct genomic segments, generally thought to be present as one copy each. The phenomenon was first revealed structurally through cryo-ET studies, which established that the eight vRNPs adopt a characteristic ‘7 + 1’ configuration within mature virions, with one central vRNP surrounded by seven peripheral ones [86] (Figure 5A). Subsequent studies further revealed inter-vRNP bridging densities through three-dimensional reconstruction of the RNP bundle in virion, suggesting the existence of direct inter-segment interactions (Figure 5B). Notably, the reconstructed complex exhibited four longer and four shorter vRNPs with distinctly different lengths, a distribution consistent with the known size difference of the eight genomic segments [28] (Figure 5C). The same overall geometry has been observed in both spherical and filamentous particles and appears to be conserved across influenza virus types. Influenza C and D viruses, despite having only seven genomic segments, have likewise been reported to contain eight RNP-like densities [87]. This observation suggests that the ‘7 + 1’ arrangement may reflect a more general organizational principle rather than simply mirroring segment number.

Selective packaging depends on segment-specific signals located within the terminal coding regions and adjacent noncoding sequences at the 3′ and 5′ ends of each vRNA [27]. Reverse genetics first showed that the terminal coding sequences of the NA segment are required for its selective incorporation, providing early experimental support for the selective packaging model [88]. Subsequent studies identified analogous signals in HA, NA, NS, and the three polymerase segments PB1, PB2, and PA [89,90,91] (Figure 5D). Further dissection of the NP packaging signal revealed two functionally distinct components. A genome incorporation signal residing in the noncoding regions is sufficient for virion entry of the vRNA in which it resides, whereas a genome bundling signal in the terminal coding regions is required for co-recruitment of the complete eight-segment set [92]. Importantly, the functionality of these signals is not intrinsic to RNA sequence alone. Site-specific charged residues within NP are required for packaging signal activity, establishing that the protein composition of the RNP directly modulates packaging specificity [93].

Beyond individual packaging signals, the eight vRNPs are linked by a network of direct inter-segment RNA-RNA contacts. Biochemical and structural studies have proposed a supramolecular assembly in which the eight segments interact to form a star-like network [94]. Sequence-specific inter-segment RNA-RNA interactions, involving the packaging signal regions of multiple vRNA pairs, have been identified in both human and avian influenza A virus strains and functionally validated by mutagenesis or competition assays [95,96,97,98,99]. How this network is organized remains unresolved, and current data suggest it is neither strictly hierarchical nor fully redundant. The PB2 segment has been proposed to exert a greater influence on the incorporation of others, suggesting that some segments contribute more than others [100], while at the same time individual contacts show considerable redundancy, such that a complete genome can be assembled through several alternative routes [101,102]. A more fundamental uncertainty is whether the proposed contacts are robust, as only a few inter-segment interactions have been validated by compensatory mutagenesis [103,104].

The redundant model is supported by single-virion imaging. Multiplexed fluorescence in situ hybridization shows that packaging can proceed through alternative segment-pairing routes rather than a single obligatory pathway [102], consistent with the cooperative pairings quantified by DNA-PAINT [105]. Interaction maps generated by different methods overlap only partially, and synonymous mutations that abolish a major contact can redistribute inter-segment interactions without apparent loss of fitness [101]. However, certain elements appear to be functionally constrained. A predicted pseudoknot in the NP segment is required for both replication and packaging [106], and disruption of a long-range interaction identified by in-cell probing reduces infectivity and attenuates virulence in mice [107]. Resolving the relative contributions of redundancy and constraint will require systematic compensatory mutagenesis of the contacts already identified, rather than additional interaction mapping.

These inter-segment contacts are thought to form within the cytosolic viral inclusions in which progeny vRNPs accumulate. Notably, these inclusions assemble before inter-segment RNA-RNA interactions are established [78], suggesting that the high local vRNP concentration they generate promotes selective inter-segment pairing. Consistent with this, LIGR-seq has shown that virion-like inter-segment contacts form only when vRNP concentration is high and are reduced when inclusion formation is prevented [108].

Selective packaging cannot be explained by any single signal or pairwise interaction alone but instead emerges from the combined action of vRNP organization, segment-specific RNA elements, and inter-segment contacts. This combinatorial mechanism ensures genome integrity while retaining sufficient plasticity to accommodate the reassortment events that drive influenza virus evolution. Several questions are still unresolved, including the identity and functional significance of the central segment in the 7 + 1 arrangement, the order in which inter-segment contacts form during cytoplasmic trafficking, and the structural basis of these RNA-RNA interactions at high resolution. Structures of vRNP-vRNP assemblies would provide an important next step toward understanding how selectivity is achieved at the molecular level.

## 6. RNP-Targeting Antiviral Strategies

The vRNP comprises components that are functionally conserved across influenza A, B, C, and D viruses, and their essential roles in viral replication make the complex an attractive antiviral target [8,30,109]. Unlike the surface glycoproteins, which tolerate extensive antigenic variation, the FluPol catalytic domains, NP oligomerization interface, and cis-acting RNA elements are highly conserved, providing targets with limited tolerance for resistance mutations [30,109]. The structural advances described in the preceding sections now provide an increasingly detailed framework for structure-based drug design against these targets.

FluPol has long been a focus of antiviral development because of its essential role in viral RNA synthesis and its sequence conservation across influenza subtypes. The most successful structure-based FluPol-targeted drugs are cap-dependent endonuclease inhibitors, exemplified by baloxavir marboxil, which blocks cap-snatching by inhibiting the PA endonuclease domain [6,29,110,111]. Crystal structures of PA in complex with baloxavir have revealed the molecular basis for inhibition [112], but the rapid emergence of resistance mutations such as PA I38T/F/N/M has reduced its clinical durability [112,113,114]. Although the variant I38T carry a fitness cost, competitive-mixture studies in ferrets demonstrate that they retain the capacity to replicate and transmit [115]. Other regions of the FluPol also present therapeutic opportunities. Guided by structures of the PB2 cap-binding domain, compounds occupying this pocket competitively block host mRNA cap recognition [116,117]. As with the endonuclease inhibitors, resistance has been observed. Substitutions in the PB2 cap-binding and adjacent linker regions, most notably at residues S324 and N510, can confer more than tenfold resistance to these compounds [118,119]. Additional targets within the polymerase complex have also been explored, including the PB1 catalytic center, where nucleoside analogues interfere with RNA chain elongation [120], and the PA-PB1 interface, where structure-guided inhibitors can abolish polymerase assembly [121]. These directions remain at earlier stages of development than the endonuclease and cap-binding inhibitors.

NP provides a second major antiviral target owing to its high conservation and essential roles in vRNP assembly and stability [30]. Current strategies have focused on disrupting NP oligomerization, interfering with RNA binding, and blocking nuclear trafficking [122,123,124,125]. Small molecules targeting the tail loop-pocket interface can induce aberrant NP assemblies or block normal NP-NP interactions [122,123], and structural analysis of intact vRNPs has also enabled identification of lead compounds against this site [26]. Screening of the RNA-binding groove has also yielded candidate inhibitors [125]. Although NP-directed compounds are still at an early stage of development, the increasing structural determination of the vRNP should facilitate structure-guided optimization [30,124].

Antisense oligonucleotides and siRNAs offer a mechanistically distinct approach by directly targeting viral RNA rather than viral proteins. Depending on their design, these agents can promote RNase H-mediated degradation or act through steric blockade and can be rapidly designed against conserved sequences [126,127]. The highly conserved noncoding regions and packaging signals at segment termini are attractive targets, as they exhibit low tolerance for escape mutations. Selection of target sites on the basis of vRNA secondary structure and sequence conservation, with preference for accessible regions, has guided the development of effective antisense agents [128,129,130]; locked nucleic acid (LNA) oligonucleotides targeting packaging stem-loop in the PB2 segment, identified through this approach, conferred complete protection against a lethal inoculum in mice [131]. Nucleic acid therapeutics and small molecule inhibitors act at complementary levels, although delivery, stability, and off-target effects continue to pose substantial challenges [132]. High-resolution structural characterization of intact vRNPs is expected help identify additional druggable sites, including conserved protein–protein interfaces and functionally critical RNA structural elements that are not currently accessible to rational design.

An alternative strategy is to target host factors that the virus depends on for its replication cycle. Targeting host factors offers a higher barrier to viral resistance, although the potential for toxicity requires careful evaluation. The best-validated example is the Raf/MEK/ERK pathway. Inhibition of MEK was first shown to reduce influenza virus titres in cell culture [133,134], and one such compound, zapnometinib (ATR-002), has demonstrated antiviral activity in preclinical models. Structure-based approaches have also yielded inhibitors of host enzymes required for viral replication. A small-molecule inhibitor of the host cap 2′-O-methyltransferase MTr1 (CMTR1), trifluoromethyl-tubercidin (TFMT), was identified by virtual screening against the MTr1 crystal structure and restricts influenza virus replication in human lung explants and in mice [135]. The numerous host factors implicated in vRNP nuclear import, export, cytoplasmic transport, and genome replication represent additional candidates whose therapeutic potential remains largely unexplored. As high-resolution structures of additional host-RNP complexes become available, they are expected to reveal further druggable sites suitable for structure-guided inhibitor design.

## 7. Conclusions and Perspectives

The influenza vRNP is now understood at a level of structural detail that would have been inconceivable when early electron micrographs first revealed its helical organization [9]. High-resolution mini-vRNP structures have defined the FluPol-NP-RNA interface, full-length reconstitutions have established the anti-parallel double-helical architecture, and in situ imaging has visualized vRNPs in transit within infected cells [14,25,26]. Together, these advances provide a coherent structural framework for RNP assembly, RNA synthesis, intracellular trafficking, and genome packaging, and have begun to inform antiviral development in a mechanism-guided manner.

However, a number of fundamental challenges persist. Near-atomic resolution insights obtained from mini-vRNPs have not yet been extended to intact complexes, leaving the precise organization of FluPol-NP-RNA contacts along the native helical scaffold undefined. Similarly, while the global features of processive strand-sliding are increasingly well supported, its molecular implementation is still poorly resolved, including how the template is recycled to preserve scaffold integrity during successive rounds of synthesis, how distinct conformational states are coordinated across transcription and replication, and how the replicase-encapsidase-ANP32 machinery is spatially arranged relative to the parental RNP. For trafficking and genome packaging, the lack of high-resolution structures of vRNP-host assemblies and inter-segment RNA-RNA interactions within intact complexes continues to limit mechanistic interpretation, particularly with respect to whether segment-specific conformations contribute to packaging selectivity. Addressing these questions will require methodological advances capable of capturing transient intermediates and resolving conformational heterogeneity in native contexts.

Single-particle cryo-EM and cryo-ET have provided complementary views of vRNP structure throughout this review. Single-particle cryo-EM achieves near-atomic resolution for relatively homogeneous specimens, including FluPol, NP-RNA filaments, mini-vRNPs, and the helical region of full-length vRNPs [19,20,23,24,25,26], but flexible or compositionally heterogeneous regions such as the polymerase-containing terminus remain poorly resolved. Cryo-ET can image vRNPs in situ within purified samples, infected cells, or intact virions, preserving near-native organization, but resolution is constrained by sample thickness, the missing wedge, and limited data throughput, typically reaching 20–30 Å for subtomogram averages of vRNPs [14,26,28,48], insufficient for characterizing molecular interfaces. Bridging these two scales will be essential for future progress. Emerging developments in cryo-ET, including improved direct detectors, optimized tilt schemes, and machine-learning-based particle identification, are steadily improving the resolution of in situ subtomogram averaging [136]. In parallel, in situ single-particle approaches offer a route to near-atomic resolution without the limitations of tilt-series acquisition [137,138]. For single-particle cryo-EM, extending high-resolution analysis from the shortest segment to longer, more flexible vRNPs remains an important goal and will require advances in both sample preparation and computational handling of conformational heterogeneity. Together, these developments raise the prospect of characterizing full-length vRNP architecture, replication intermediates, host-RNP complexes, and inter-segment contacts at molecular detail directly within infected cells.

Structure-based antiviral development has already demonstrated the translational potential of targeting the vRNP. The clinical success of baloxavir marboxil establishes the vRNP as a tractable source of druggable targets, while structural analyses of resistance mutations are guiding the design of next-generation inhibitors [110,111,112]. Beyond small-molecule inhibitors, the increasing development of nucleic acid-based therapeutics further highlights the potential of targeting conserved and functional RNA elements within the vRNP [128,129,130,131]. As higher-resolution structures of intact vRNPs become available, they will enable the identification of both critical interaction interfaces and functional RNA elements, thereby enabling more precise and comprehensive antiviral design.

## Data Availability

No new data were created or analyzed in this study. Data sharing is not applicable to this article.
